# Eye-tracking Analysis of Interactive 3D Geovisualization

**DOI:** 10.16910/jemr.10.3.2

**Published:** 2017-05-31

**Authors:** Lukas Herman, Stanislav Popelka, Vendula Hejlova

**Affiliations:** Masaryk University, Czech Republic; PalackÝ University, Czech Republic

**Keywords:** eye-tracking, 3D visualization, 3D model, cartography, Geographic Information System, 3D analysis tool

## Abstract

This paper describes a new tool for eye-tracking data and their analysis with the use of interactive 3D models. This tool helps to analyse interactive 3D models easier than by time-consuming, frame-by-frame investigation of captured screen recordings with superimposed scanpaths. The main function of this tool, called 3DgazeR, is to calculate 3D coordinates (X, Y, Z coordinates of the 3D scene) for individual points of view. These 3D coordinates can be calculated from the values of the position and orientation of a virtual camera and the 2D coordinates of the gaze upon the screen. The functionality of 3DgazeR is introduced in a case study example using Digital Elevation Models as stimuli. The purpose of the case study was to verify the functionality of the tool and discover the most suitable visualization methods for geographic 3D models. Five selected methods are presented in the results section of the paper. Most of the output was created in a Geographic Information System. 3DgazeR works with the SMI eye-tracker and the low-cost EyeTribe tracker connected with open source application OGAMA, and can compute 3D coordinates from raw data and fixations.

## Introduction

The introduction summarizes state of the art in 3D cartography eye-tracking research, followed by a presentation of previous attempts to record eye-tracking data over interactive 3D models. In the methods section, 3DgazeR and its implementation are described. The results contain five selected data visualization methods applied in the example of the simple case study. At the end of the paper, a summary of 3DgazeR advantages and limitations is described.

### 3D Geovisualization

Bleisch ( 1 ) defines 3D geovisualization as a generic term used for a range of 3D visualizations representing the real world, parts of the real world, or other data with a spatial reference. With the advent of virtual globes such as Google Earth, or perhaps even earlier with the notion of a digital earth ( 2 ), they have become increasingly popular, and many people already know about 3D geovisualizations even though they may not call them as such. Most 3D geovisualizations are digital elevation models draped with ortho or satellite imagery and relatively detailed 3D city models ( 1 ). These perspective views are often referred to as *3D maps*. The overview of the usability and usefulness of 3D geovisualizations was presented by Çöltekin, Lokka ( 3 ). Authors categorized the results from existing empirical studies according to visualization type, task type, and user type.

3D geovisualization is not limited to the depiction of terrain where the Z axis represents elevation. The development of a phenomenon in time is often displayed, for example, with the aid of a so-called Space-Time-Cube (STC). Hägerstraand ( 4 ) proposed a framework for time geography to study social interaction and the movement of individuals in space and time. The STC is a visual representation of this framework where the cube’s horizontal plane represents space, and the 3D vertical axis represents time ( 5 ). With a Space-Time-Cube, any spatio-temporal data can be displayed. That data can be, for example, information recorded by GPS devices, statistics with location and time components, or data acquired with eye-tracking technology ( 6 ).

3D maps and visualizations can generally be divided into two categories: static and interactive. Static visualizations are essentially perspective views (images) of any 3D scene. In interactive 3D visualizations, the user can control and manipulate the scene. The disadvantages of static 3D maps are mainly overlapping objects in the 3D scene and the distortion of distant objects. Inexperienced users could have problems with scene manipulation using a mouse ( 7 ).

Most of the cases referred to as 3D geovisualization are not true 3D, but a pseudo 3D (or 2.5D – each X and Y coordinate corresponds to exactly one Z value). According to Kraak ( 8 ), true 3D can be used in those cases where special equipment achieves realistic 3D projection (i.e. 3D LCD displays, holograms, stereoscopic images, anaglyphs or physical models).

Haeberling ( 9 ) notes that there is almost no cartographic theory or principles for creating 3D maps. In his dissertation, Góralski ( 10 ) also argues that solid knowledge of 3D cartography is still missing. A similar view can be found in other studies ( 7, 11-13 ). The authors report that there is still very little known about how and in which cases 3D visualization can be effectively used. Performing an appropriate assessment of the usability of 3D maps is necessary.

### Usability methods for 3D geovisualization (3D maps)

Due to the massive increase in map production in recent years, it is important to focus on map usability research. Maps can be modified and optimized to better serve users based on the results of this research.

One of the first works dealing with map usability research was published by Petchenik ( 14 ). In her work "Cognition in Cartography", she states that for the successful transfer of information between the map creator and map reader, it is necessary for the reader to understand the map in the same way as the map creator. The challenge of cognitive cartography is understanding how users read various map elements and how the meanings of those elements between different users vary.

The primary direction of cognitive cartography research leads to studies in how maps are perceived, to increase their efficiency, and adapt their design to the needs of a specific group of users. The International Cartographic Association (ICA) has two commissions devoted to map users, the appraisal of map effectiveness, and map optimization – the Commission on Use and User Issues ( http://use.icaci.org/ ) and the Commission on Cognitive Visualization ( http://cogvis.icaci.org/ ). User aspects are examined in respect of the different purposes of maps (for example Staněk, Friedmannová ( 15 ) or Kubíček, Šašinka ( 16 ).

Haeberling ( 17 ) evaluated the design variables employed in 3D maps (camera angle and distance, the direction of light, sky settings and the amount of haze). Petrovič and Mašera ( 18 ) used a questionnaire to determine user preferences between 2D and 3D maps. Participants of their study had to decide which type of map they would use to solve four tasks: measuring distances, comparing elevation, determining the direction of north, and evaluating the direction of tilt. Results of the study of Petrovič and Mašera ( 18 ) showed that 3D maps are better for estimating elevation and orientation than their 2D equivalents, but 3D maps may cause potential problems for distance measuring.

Savage, Wiebe ( 19 ) tried to answer the question whether using 3D perspective views has an advantage over using traditional 2D topographic maps. Participants were randomly divided into two groups and asked to solve spatial tasks with a 2D or a 3D map. The results of the study showed no advantage in using 3D maps for tasks that involved estimating elevation. Additionally, in tasks where it was not necessary to determine an object’s elevation (e.g. measuring distances), the 3D variant was not as good.

User testing of 3D interactive virtual environments is relatively scarce. One of the few articles describing such an environment is presented by Wilkening and Fabrikant ( 20 ). Using the Google Earth application, they monitored the proportion of applied movement types – zoom, pan, tilt, and rotation. Bleisch, Burkhard ( 21 ) assessed the 3D visualization of abstract numeric data. Although speed and accuracy were measured, no information about navigation in 3D space was recorded in this study. Lokka and Çöltekin ( 22 ) investigated memory capacity in the context of navigating a path in a virtual 3D environment. They observed the differences between age groups.

Previous studies ( 20, 23, 24 ) indicate that there are considerable differences between individuals in how they read maps, especially in the strategies and procedures used to determine an answer to a question. To understand map reading strategy, the use of eye-tracking facilitates the study.

### Eye-tracking in Cartography

Although eye-tracking to study maps was first used in the late 1950s, it has seen increased use over the last ten to fifteen years. Probably the first eye-tracking study for evaluating cartographic products was the study of Enoch ( 25 ), who used as stimuli simple maps drawn on a background of aerial images. Steinke ( 26 ) presented one of the first published summaries about the application of eyetracking in cartography. He compiled the results of former research and highlighted the importance of distinguishing between the perceptions of user groups of different age or education.

Today, several departments in Europe and the USA conduct eye-tracking research in cartography ( 27 ). In Olomouc, Czech Republic, eye-tracking has been used to study the output of landscape visibility analyses ( 28 ) and to investigate cartographic principles ( 29 ). In Zurich, Switzerland, Fabrikant, Rebich-Hespanha ( 30 ) evaluated a series of maps expressing the evolution of phenomenon over time and weather maps ( 31 ). Çöltekin from the same university analyzed users’ visual analytics strategies ( 32 ). In Ghent, Belgium paper and digital topographic maps were compared ( 33 ) and differences in attentive behavior between novice and expert map users were analyzed ( 34 ). Ooms, Coltekin ( 35 ) proposed the methodology for combining eye-tracking with user logging to reference eyemovement data to geographic objects. This approach is similar to ours, but instead of 3D model a dynamic map is used.

### Eye-tracking to assess 3D visualization

The issue of 3D visualization on maps has so far only been addressed marginally. At the State University of Texas, Fuhrmann, Komogortsev ( 36 ) evaluated the differences in how a traditional topographic map and its 3D holographic equivalent were perceived. Participants were asked to suggest an optimal route. Analysis of the eyetracking metrics showed the better option to be the holographic map.

One of the first and more complex studies dealing with eye-tracking and the evaluation of 3D maps is the study by Putto, Kettunen ( 37 ). In this study, the impact of three types of terrain visualization was evaluated while being required to solve four tasks (visual search, area selection, and route planning). The shortest average length of fixation was observed for the shaded relief, indicating that this method is the easiest for users.

Eye-tracking for evaluating 3D visualization in cartography is widely used at Palacký University in Olomouc, Czech Republic, with studies examining the differences in how 3D relief maps are perceived ( 38 ), 3D maps of cities ( 39 ), a 3D model of an extinct village ( 40 ), and tourist maps with hill-shading ( 41 ) being produced there. These studies showed that it is not possible to generalize the results and state that 3D is more effective than 2D or vice versa. The effectivity of visualization depends on the exact type of stimuli and also on the task.

In all these studies static images were used as stimuli. Nevertheless, the main advantage of 3D models is being able to manipulate them (pan, zoom, rotate). An analysis of eye-tracking data measured on interactive stimuli is costly, as eye-trackers produce video material with overlaid gaze-cursors and any classification of fixations requires extensive manual effort ( 42 ). Eye tracking studies dealing with interactive 3D stimuli typically comprise a time-consuming frame-by-frame analysis of captured screen recordings with superimposed scanpaths. One of the few available gaze visualization techniques for 3D contexts is the representation of fixations and saccades as 3D scanpaths ( 43 ). A challenge with 3D stimuli is mapping fixations onto the correct geometrical model of the stimulus ( 44 ).

Several attempts to analyze eye-tracking data recorded during the work with interactive 3D stimuli exist. Probably the most extensive work has been done by Stellmach, who developed tool called SWEETER – a gaze analysis tool adapted to the Tobii eye-tracker system and XNA Framework. SWEETER offers a coherent framework for loading 3D scenes and corresponding gaze data logs, as well as deploying adapted gaze visualizations techniques ( 45 ).

Another method for visualizing the gaze data of dynamic stimuli was developed by Ramloll, Trepagnier ( 46 ). It is especially useful for 3D objects on retail sites allowing shoppers to examine products as interactive, non-stereoscopic 3D objects on 2D displays. In this approach, each gaze position and fixation point is mapped to a 3D object’s relevant polygon. A 3D object is then flattened and overlaid with the appropriate gaze visualizations. The advantage of this flattening is that the output can be reproduced on a 2D static medium (i.e. paper).

Both approaches used a remote eye-tracker to record data. Pfeiffer ( 42 ) used a head-mounted eye-tracking system by Arrington Research. This study extended recent approaches of combining eye-tracking with motion capture, including holistic estimations of the 3D point of regard. In addition, he presented a refined version of 3D attention volumes for representing and visualizing attention in 3D space.

Duchowski, Medlin ( 47 ) developed an algorithm for binocular eye-tracking in virtual reality, which is capable of calculating the three-dimensional virtual coordinates of the viewer’s gaze.

A head-mounted eye-tracker from the SMI was used in the study of Baldauf, Fröhlich ( 48 ), who developed the application KIBITZER – a wearable gaze-sensitive system to explore urban surroundings. The eye-tracker is connected via a smartphone and the user’s eye-gaze is analyzed to scan the visible surroundings for georeferenced digital information. The user is informed about points of interest in his or her current gaze direction.

SMI glasses were also involved in the work of Paletta, Santner ( 49 ), who used them in combination with Microsoft Kinect. A 3D model of the environment was acquired with Microsoft Kinect and gaze positions captured by the SMI glasses were mapped onto the 3D model.

Unfortunately, all the presented approaches work with specific types of device and are not generally available for the public. For this reason, we decided to develop our own application called 3DgazeR (3D Gaze Recorder). 3DgazeR can place recorded raw data and fixations into the 3D model’s coordinate system. The application works primarily with geographical 3D models (DEM – Digital Elevation Models in our pilot study). Majority of visualization of results of the case study is performed in open source Geographic Information System QGIS. The application works with data from an SMI RED 250 device and a low-cost, EyeTribe eye-tracker. This eye-tracker is connected with open source application OGAMA. Many different eye-trackers could be connected with OGAMA and then our tool will work with their data.

## Methods

**Fig. 1:**
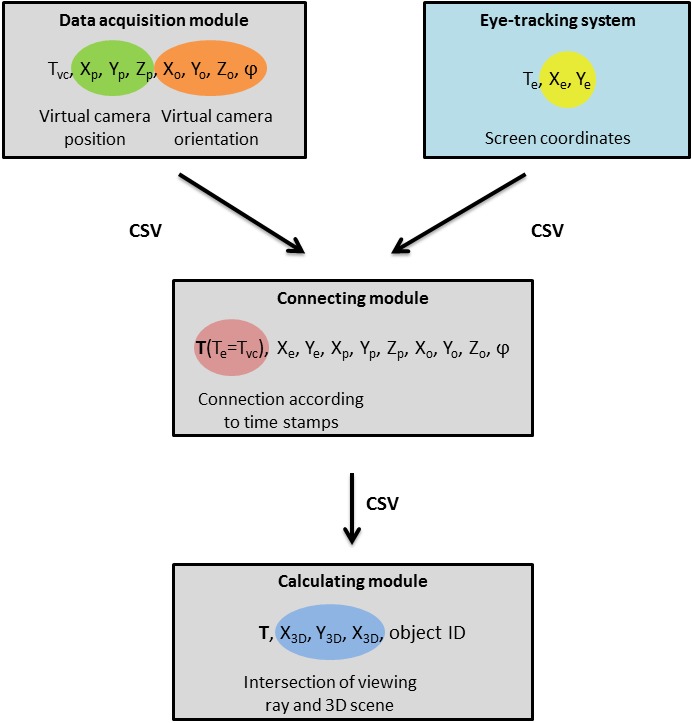
Schema of 3DgazeR modules.

We designed and implemented our own experimental application, 3DgazeR, due to the unavailability of tools allowing eye-tracking while using interactive 3D stimuli. The main function of this instrument is to calculate the 3D coordinates (X, Y, Z coordinates of the 3D scene) for individual points of view. These 3D coordinates can be calculated from the values of the position and orientation of a virtual camera and the 2D coordinates of the gaze on the screen. 2D screen coordinates are obtained from the eyetracking system, and the position and orientation of the virtual camera are recorded with the 3DgazeR tool (see Figure 1).

3DgazeR incorporates a modular design. The three modules are:

• **Data acquisition module**

• **Connecting module** to combine the virtual camera data and eye-tracking system data

• **Calculating module** to calculate 3D coordinates

The modular design reduces computational complexity for data acquisition. Data for gaze position and virtual camera position and orientation are recorded independently. Combining the data and calculating 3D coordinates is done in the post-processing phase. Splitting the modules for combining data and calculating 3D coordinates allows information from different eye-tracking systems (SMI RED, EyeTribe) and various types of data (raw data, fixation) to be processed.

All three modules constituting 3DgazeR only use open web technologies: HTML (HyperText Markup Language), PHP (Hypertext Preprocessor), JavaScript, jQuery and JavaScript library for rendering 3D graphics X3DOM. Library X3DOM was chosen because of its broad support in commonly used web browsers, as well as documentation for the accessibility and availability of software to create stimuli. X3DOM uses an X3D (eXtensible 3D) structure format and is built on HTML5, JavaScript, and WebGL. The current implementation of X3DOM uses a so-called fallback model that renders 3D scenes through an InstantReality plug-in, a Flash11 plug-in, or WebGL. To run X3DOM, no specific plug-in is needed. X3DOM is free for both for non-commercial and commercial use ( 50 ). Common JavaScript events, such as *onclick* on 3D objects, are supported in X3DOM. A runtime API is also available and provides a proxy object for reading and modifying runtime parameters programmatically. The API functions serve for interactive navigation, resetting views or changing navigation modes. X3D data can be stored in an HTML file or as part of external files. Their combination is achieved via an inline element. Particular X3D elements can be clearly distinguished through their DEF attribute, which is a unique identifier. Other principles and advantages of X3DOM are described in ( 50-53 ).

### Data acquisition module

The data acquisition module is used to collect primary data. Its main component is a window containing the 3D model used as a stimulus. This 3D scene can be navigated or otherwise manipulated. The rendering of virtual content inside a graphics pipeline is the orthographic or perspective projection of 3D geometry onto a 2D plane. The parameters for this projection are usually defined by some form of virtual camera. Only main parameters, position, and orientation of the virtual camera are recorded in the proposed solution. The position and orientation of the virtual camera are recorded every 50 milliseconds (frequency of records 20 Hz). The recording is performed using functions from X3DOM runtime API and JavaScript in general. The recorded position and orientation of the virtual camera is sent every two seconds to a server and stored using a PHP script to a CSV (Comma Separated Value) file. Storage of the 3D scene loading time is necessary for subsequent combination with eye-tracking data. Similarly, termination of the 3D scene is also stored. The interface is designed as a full-screen 3D scene while input for answers is provided on the following screen (after the 3D scene).

### Connection module

The connecting module combines two partial CSV files based on timestamps. The first step is joining trimmed data (from the eye-tracker and from the movement of the virtual environment) by markers of the beginning and end of the depiction of a 3D scene. The beginning in both records is designated as time 0. Each record from the eye-tracker is then assigned to the nearest previous recorded position of the virtual camera (by timestamp), which is the simplest method of joining temporal data and it was not difficult to implement. The maximum time deviation (inaccuracy) is 16.67 ms.

**Fig. 2:**
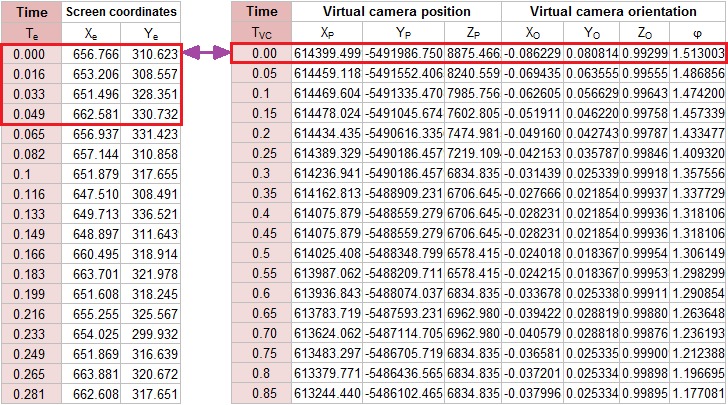
Examples of data about eye-tracking data (left) and virtual camera movement (right) and schema of their connecting

Four variants of the connecting module were created – for SMI RED 250 and EyeTribe, and for raw data and fixations. The entire connecting module is implemented in JavaScript.

### Calculating module

**Fig. 3:**
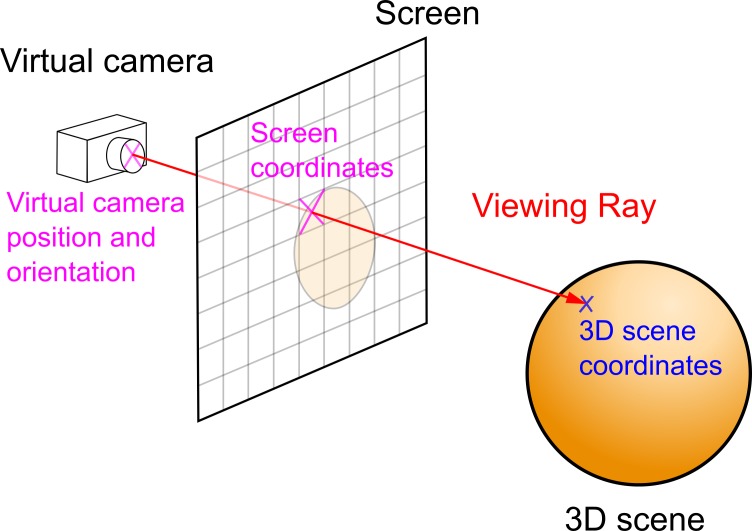
Principle of ray casting method for 3D scene coordinates calculation.

The calculating module comprises a similar window and 3D model to those used in the test module. The same screen resolution must be used as during the acquisition of data. For every record, the intersection of the viewing ray with the displayed 3D model is calculated. A 3D scene is depicted with a virtual camera’s input position and orientation. The X3DOM runtime API function *getViewingRay* and screen coordinates as input data are used for this calculation. Setting and calculating the virtual camera’s parameters is automated using the FOR cycle. The result is a table containing timestamps, 3D scene coordinates (X, Y, Z), the DEF element the ray intersects with, and optionally, a normal vector to this intersection. If the user is not looking at any particular 3D object, this fact is also recorded, including whether the user is looking beyond the dimensions of the monitor. This function is based on ray casting method (see Figure 3) and can be divided into three steps:

• calculation of the direction of the viewing ray from the virtual camera position, orientation and screen coordinates (function *calcViewRay*);

• ray casting to the scene;

• finding the intersection with the closest object (function *hitPnt*).

For more information about ray casting see Hughes, Van Dam ( 54 ).

For additional processing, analysis, and visualization of calculated data, GIS software is used. It was primarily open source program QGIS, but ArcGIS with 3D Analyst and ArcScene (3D viewing application) can also be used. We worked with QGIS version 2.12 with several additional plug-ins. Most important was Qgis2threejs plug-in. Qgis2threejs creates 3D models and exports terrain data, map canvas images, and overlaid vector data to a web browser supporting WebGL).

## Pilot study

Our pilot experiment was designed as exploratory research. The primary goal of this experiment was to test the possibilities of 3DgazeR in evaluating different methods of visualization and analyzing eye-tracking data acquired with an interactive 3D model.

### Apparatus, tasks and stimuli

For the testing, we chose a low-cost EyeTribe device. Currently, the EyeTribe tracker is the most inexpensive commercial eye-tracker in the world at a price of $99 ( https://theeyetribe.com) Popelka, Stachon ( 55 ) compared the precision of the EyeTribe and the professional device SMI RED 250. The results of the comparison show that the EyeTribe tracker can be a valuable tool for cartographic research. The eye-tracker was connected to OGAMA software ( 56 ). The device operated at a frequency of 30Hz. EyeTribe also works with a frequency of 60Hz, however saving information about camera orientation caused problems with a frequencies higher than 20Hz. Some computer setups were not able to store camera data correctly when the frequency was higher than 20Hz. The length of the file was shorter than real recording, because some rows were omitted.

**Fig. 4:**
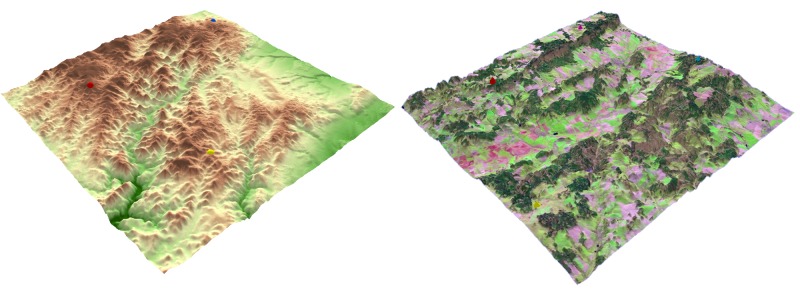
An example of stimuli from variant A – terrain covered with a hypsometric scale (left) and variant B – terrain covered with a satellite image (right).

Two versions of the test were created – variant A and variant B. Each variant included eight tasks over almost the same 3D models (differing only in the used texture). The 3D models in variant A had no transparency, and the terrain was covered with a hypsometric color scale (from green to brown). The same hypsometric scale covered four 3D models in variant B, but transparency was set at 30%. The second half of the models in variant B had no transparency, but the terrain was covered with satellite images from Landsat 8. The order of the tasks was different in both variants. A comparison of variant A and variant B for the same task is shown in Figure 4. Four tasks were required:

• Which object has the highest elevation? (Variant A – tasks 1, 5; Variant B – tasks 1, 2)

• Find the highest peak. (Variant A – tasks 2, 6; Variant B – tasks 3, 4)

• Which elements are visible from the given position? (Variant A – tasks 3, 7; Variant B – tasks 5, 6)

• From which positions a given object is visible? (Variant A – tasks 4, 8; Variant B – tasks 7, 8)

The first two tasks had only one correct answer, while the other two had one or more correct answers.

### Design and participants

Before the pilot test we decided that 20 participants would be tested on both variants, with an interval of at least three days between both testing sessions. Participants were recorded on both variants, but not influenced by a learning effect when performing the second variant of the test because of the interval.

Half of the participants were students of the Department of Geoinformatics with cartographic knowledge, half of them were cartographic novices. Half of the participants were men, half women. The age range was 18-32 years.

Screen resolution was 1600 x 900 and the sampling frequency was set to 30 Hz. Each participant was seated at an appropriate distance from the monitor with an eye-tracking device calibrated with 16 points. Calibration results of either Perfect or Good (on the scale used in OGAMA) were accepted. An external keyboard was connected to the laptop to start and end the tasks (F2 key for start and F3 for the end). A researcher controlled the keyboard. The participant performed the test using only a common PC mouse.

The experiment began with calibrating the device in the OGAMA environment. After that, participants filled in their ID and other personal information such as age, sex, etc. The experiment was captured as a screen recording.

Prepared with individual HTML pages, the experiment included questions, tasks, 3D models, and input screens for answers. The names of the CSV files where recording of the virtual camera movement would be stored coincided with the task in the subsequently created eye-tracking experiment in OGAMA. This would allow correct combination in the connecting module.

As recording began, a page with initial information about the experiment appeared. The experiment ran in Google Chrome in full-screen mode and is available (in Czech) at http://eyetracking.upol.cz/3d/. Each task was limited to 60 seconds duration, and the whole experiment lasts approximately 10 to 15 minutes. Longer total time of the experiment may affect the user performance. From the evidence from previous experiments, we found out that when the recording is longer than e.g. 20 minutes, the participants started to be tired and they lose concentration.

Care was taken with the correct starting time for tasks. A screen with a target symbol appeared after the 3D model had loaded. The participant switched to the task by pressing the F2 key. This keypress was recorded by OGAMA and used by 3DGazeR to divide recording according to the task. After that, a participant could manipulate the 3D model to try to discover the correct answer. The participant then pressed F3, and a screen with a selection of answers appeared.

### Recording, data processing and validation

It is necessary to store the data for each task separately, alternatively control or manually modify (e.g. delete the unnecessary lines at the end of recording). The data is then processed in the connecting module where data from the eyetracking device is combined with virtual camera movement. The output is then sent to the calculation module which must be switched to full-screen mode. The calculation must take place on the same screen resolution as the testing. The output should be modified for import into GIS software and visualized. For example, the data format of the time column had to be modified into a form required to subsequently create animation.

**Fig. 5:**
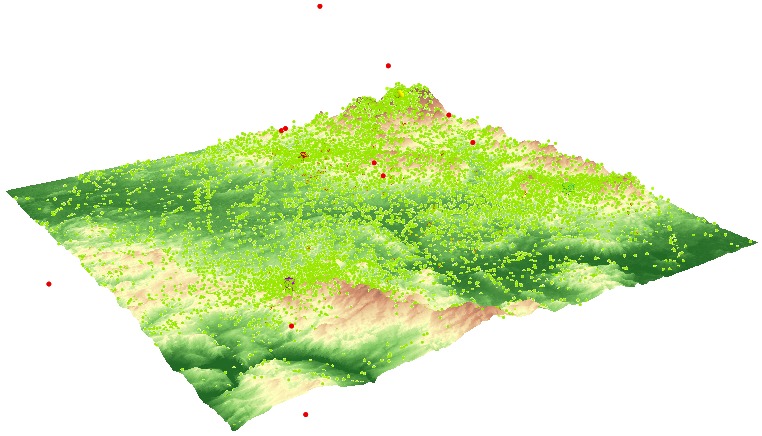
Raw data displayed as a layer in GIS-software (green points – calculated 3D gaze data; red – points with incorrect el-evation).

These adjusted data can be imported into QGIS. CSV data are loaded and displayed here using the Create a Layer from a Delimited Text File dialog. The retrieved data can be stored in GML (Geography Markup Language) or Shapefile as point layers. After the export and re-render of this new layer above the 3D model, it is possible that some data may have the wrong elevation (see Figure 5). This distortion occurs when the 3D model is rotated while eyes are simultaneously focused on a specific place, or when the model is rotated, and eyes track with a smooth pursuit. To remove these distortions and correctly fit eye-tracking data exactly on the model, the Point Sampling Tool plug-in in Qgis2threejs was used.

### Evaluation of the data validity

For the evaluation of the validity of 3DGazeR output, we have created the short animation of 3D model with one red sphere in the middle. The diameter of the sphere was approximately 1/12 of the 3D model width. In the beginning, the sphere was located in the middle of the screen. After five seconds, the camera changed its position (it took two seconds), and the sphere moved to the upper left side of the screen. The camera stayed there for six seconds and then moved again, so the sphere was displayed in the next corner of the screen. This process was repeated for all four corners of the screen. The validation study is available at http://eyetracking.upol.cz/3d/.

**Fig. 6:**
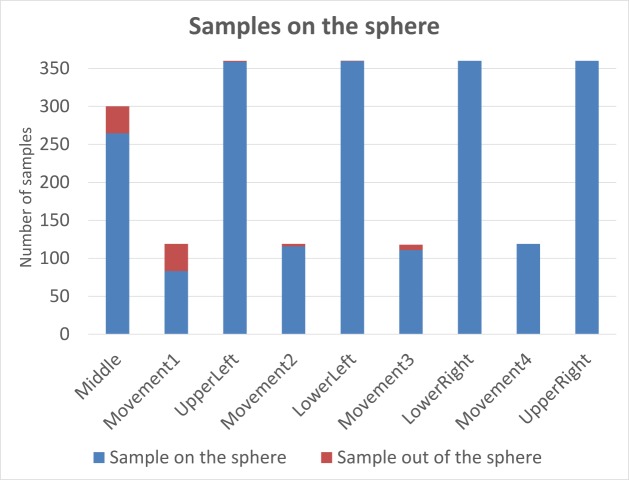
Evaluation of the data validity. Red color corresponds to the data samples where gaze was not recorded on the target sphere.

The task of the participant was to look at the sphere all time. The validation was performed on five participants. Recorded data were processed in connection and calculating modules of 3DGazeR. For the evaluation of the data validity, we decided to analyze how many data samples were assigned to the sphere. Average values for all five participants are displayed in Figure 6. Each bar in the graph represents one camera position (or movement). The blue color corresponds to the data samples where the gaze coordinates were assigned to the sphere; the red color is used when the gaze was recorded out of the sphere. It is evident that inaccuracies were observed for the first position of the sphere because it took some time to participants to find the sphere. A similar problem was found when the first movement appeared. Later, the percent of samples recorded out of the sphere is minimal. In total, average amount of samples recorded out of the sphere is 3.79 %. These results showed that the tool works correctly and the inaccuracies are caused by the inability of the respondents to keep eyes focused on the sphere that was verified by watching the video recording in OGAMA.

## Results

Visualization techniques allow researchers to analyze different levels and aspects of recorded eye tracking data in an exploratory and qualitative way. Visualization techniques help to analyze the spatio-temporal aspect of eye tracking data and the complex relationships it contains ( 44 ). We decided to use both fixations and raw data that for visualization. 3D alternatives to the usual methods of eye-tracking data were created, and other methods suitable for visualization of 3D eye-tracking data were explored. The following visualization methods were tested:

• 3D raw data

• 3D scanpath (fixations and saccades)

• 3D attention map

• Animation

• Graph of Z coordinate variation over time.

### 3D raw data

**Fig. 7:**
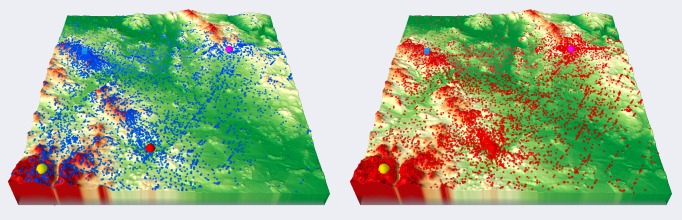
Comparison of 3D raw data (red points – females, blue points – males) for variant B, task 6.

First, we tried to visualize raw data as simple points placed on a 3D surface. This method is very simple, but its main disadvantage is the poor arrangement of depicting data in this way, mainly in areas with a high density of points. The size, color, and transparency of symbols can be set in used GIS software. With this type of visualization, data from different groups of participants can be compared, as shown in Figure 7. Raw data displayed as points were used as input for creating other types of visualizations. Figure 7 shows the 3D visualization of raw data created in QGIS. Visualization of a large number of points in the 3D scene in a web browser through Three.js is hardware demanding. Thus, visualization of raw data is more effective in ArcScene.

### 3D scanpath

The usual approach for depicting eye-tracking data is scanpath visualization superimposed on a stimulus representation. Scanpaths show the eye-movement trajectory by drawing connected lines (saccades) between subsequent fixation positions. A traditional spherical representation of fixations was chosen, but ( 45 ) also demonstrate different types of representation. Cones can be used to represent fixations or viewpoints and view directions for camera paths.

The size of each sphere was determined from the length of the fixation. Fixations were detected in OGAMA environment with the use of I-DT algorithm. The settings for thresholds were set to maximum distance of 30px and minimum number of three samples per fixation. Fixation length was used as the attribute for the size of each sphere. Transparency (30 %) was set because of overlaps. In the next step we created 3D saccades linking fixations. The PointConnector plug-in in QGIS was used for this purpose.

**Fig. 8:**
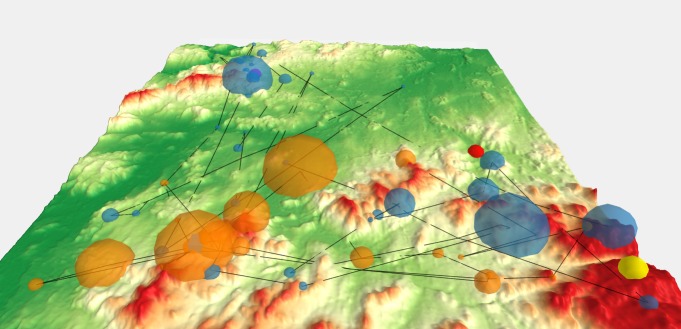
Scanpath (3D fixations and saccades) of one user for variant A, task 4. Interactive version is available at http://eyetracking.upol.cz/3d/.

This visualization method is quite clear. It provides an overview of the duration of individual fixations, their position, and relation to each other. It tells where the participant’s gaze lingered and where it stayed only briefly. Lines indicate if a participant skipped between remote locations and back or if the observation of the stimulus was smooth. The scanpath from one participant solving variant A, task 4 is shown in Figure 8. From the length of fixations, it is evident that the participant observed locations near spherical bodies defining the target points crucial for task solving. His gaze shifted progressively from target to target, whereby the red target attracted the most attention.

### 3D Attention Map

Visual gaze analysis in three-dimensional virtual environments still lacks the methods and techniques for aggregating attentional representations. Stellmach, Nacke ( 45 ) introduced three types of attention maps suitable for 3D stimuli – projected, object-based, and surface-based attention maps. In Digital Elevation Models, the use of projected attention maps is the most appropriate. Object-based attention maps, which are relatively similar to the concept of Areas of Interest, can also be used for eye-tracking analysis of interactive 3D models with 3DgazeR. In this case, stimuli must contain predetermined components (objects) with unique identifiers (attribute DEF in X3DOM library).

**Fig. 9:**
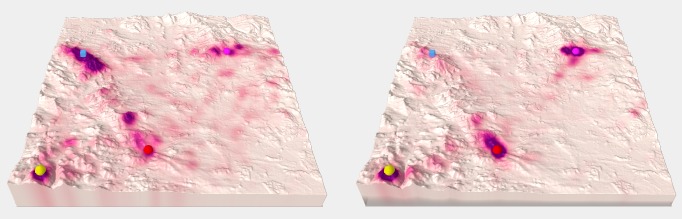
Comparison of 3D attention maps from cartographers (left) and non-cartographers (right) for variant B, task 6. Inter-active versions are available at http://eyetracking.upol.cz/3d/.

Projected attention maps can be created in the ArcScene environment using the Heatmap plug-in in QGIS function. Heatmap calculates the density of features (in our case fixations) in a neighborhood around those features. Conceptually, a smoothly curved surface is fitted over each point. The important factors for creating Heatmap are grid cell size and search radius. We used a cell size of 25 m (it is about one thousandth of the terrain model size) and search radius as an implicit value (see Figure 9).

The advantage of projected attention maps is their clarity for visualization of a large amount of data. In a Geographic Information System, the exact color scheme of the attention map can be defined (with minimum and maximum values).

An interesting result was obtained from task 6, variant B. Figure 9 compares the resultant attention maps from participants with cartographic knowledge with those from the general public. For cartographers, the most important part of the terrain was around the blue cube. Participants without cartographic knowledge focused on other objects in the terrain. An interpretation of this behavior could be that the cartographers were consistent with the task and looked at the blue cube from different areas. By contrast, novices used the opposite approach and investigated which objects were visible from the blue cube’s position.

### Animation

A suitable tool for evaluating user strategies is animation. Creating an animation with a 3D model is not possible in QGIS software, so we used ArcScene (with the function Create Time Animation) for this purpose. The model can also be rotated during the animation, providing interactivity from data acquisition through to final analysis. Animations can be used to study fixations of individuals or to compare several users. Animations can be exported from ArcScene software as video files (e.g. AVI), but it loses its interactivity. AVI files exported from ArcScene are available at http://eyetracking.upol.cz/3d/. A similar method to animation is taking screenshots, which can also be alternatively used in the qualitative (manual) analysis of critical task solving moments, such as at the end or when entering an answer.

### Graph

**Fig. 10:**
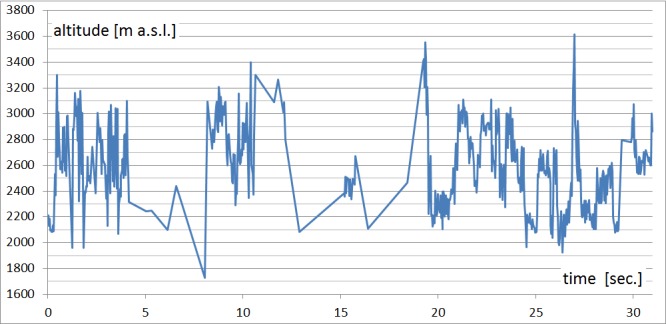
Graph of observed elevations during task (variant A, task 4, participant no. 20).

When analyzing 3D eye-tracking data, it would be appropriate to concentrate on analyzing the Z coordinate (height). From the data recorded with 3DgazeR, the Z coordinate’s changes over time can be displayed, so the elevations the participants looked at in the model during the test can be investigated. Data from the program ArcScene were exported into a DBF table and then analyzed in OpenOffice Calc. A scatter plot with data points connected by lines should be used here. A graph for one participant (see Figure 10) or multiple participants can be created. A graph of Z coordinate raw data values was created in this case.

It is apparent from this graph when participants looked at higher ground or lowlands. In Figure 10, we can see how the participants initially fluctuated between elevations in observing locations and focused on the highest point around the 27^th^ second during the task. In general, we con-clude that this participant studied the entire terrain quite carefully and looked at a variety of low to very high eleva-tions.

## Discussion

We developed our own testing tool, 3DgazeR, because none of the software tools found through literature review were freely available for application. Those software tools worked with specific devices, or had proprietary licenses, and were not free or open source software. 3DgazeR is freely available to interested parties under a BSD license to fill this gap. English version of 3DgazeR is available at http://eyetracking.upol.cz/3d/. Furthermore, 3DgazeR has several significant advantages:

• It permits evaluation of different types of 3D stimuli because the X3DOM library is very flexible – for an overview of various 3D models displayed through X3DOM see ( 51-53 )

• It is based on open web technologies and thus an inexpensive solution, and does not need special software installed or plug-ins on the client or server sides

• It combines open JavaScript libraries and PHP, and so may be easily extended or modified

• It writes data into a CSV file, allowing easy analysis under various commercial, freeware, and open source programs.

3DgazeR also demonstrates general approaches in creating eye-tracking analyses of interactive 3D visualizations. Some limitations of this testing tool, however, were identified during the pilot test:

• A higher recording frequency of virtual camera position and orientation in the data acquisition module would allow greater precision during analysis

• Some of the calculated 3D gaze data (points) are not correctly placed on a surface. This distortion happens when the 3D model is rotated while eyes are simultaneously focused on a specific place, or when the model is rotated, and eyes track with a smooth motion. A higher frequency in recording virtual camera position and orientation can solve this problem

• Data processing is time-consuming and involves manual effort. Automating this process and developing tools to speed up data analysis and visualization would greatly enhance its productivity.

Future development of 3DgazeR should aim at overcoming these limitations. Other possible extensions to our methodology and the 3DgazeR tool have been identified:

• We want to modify 3DgazeR to support other types of 3D models (e.g. 3D models of buildings, machines, or similar objects), and focus mainly on the design and testing of such procedures to create 3D models comprising individual parts marked with unique identifiers (as mentioned above – with a DEF attribute). Such 3D models also allow us to create object-based attention maps. The first trials in this direction are already underway. They represent simple 3D models which are predominantly created manually. This is time-consuming and requires knowledge of XML (eXtensible Markup Language) structure and X3D format. We would like to simplify and automate this process as much as possible in the future.

• We would like to increase the frequency of records of position and orientation of the virtual camera, especially during its movement. On the other hand, when it is no user interaction (virtual camera position is not changed at this time), it would be suitable to decrease the frequency to reduce the size of created CSV file. The ideal solution would be the recording with adaptive frequency, depending on whether the virtual camera is moving or not.

• We also want to improve the connecting module to use more accurate method for joining data of the movement of the virtual camera with data from the eye-tracking system.

• We tested primarily open source software (QGIS, OpenOffice Calc) for visualization of the results. Creation of 3D animation was not possible in QGIS, so commercial software ArcScene was used for this purpose. The use of ArcScene is more effective also in the case of raw data visualization. We want to test the possibilities of advanced statistical analysis in some open source program, e.g. R.

3DgazerR enables each participant’s strategy (e.g. Fig. 8 and Fig. 10) to be studied, their pairs compared, and group strategies (e.g. Fig. 7 and Fig. 9) analyzed. In the future, once the above adjustments and additions have been included, we want use 3DgazerR for complex analysis of user interaction in virtual space and compare 3D eyetracking data with user interaction recordings introduced by Herman and Stachoň ( 24 ). We would like to extend the results of existing studies, e.g. ( 45 ), in this manner.

## Conclusion

We created an experimental tool called 3DgazeR to record eye-tracking data for interactive 3D visualizations. 3DgazeR is freely available to interested parties under a BSD license. The main function of 3DgazeR is to calculate 3D coordinates (X, Y, Z coordinates of the 3D scene) for individual points of view. These 3D coordinates can be calculated from the values of the position and orientation of a virtual camera and the 2D coordinates of the gaze upon the screen. 3DgazeR works with both the SMI eye-tracker and the low-cost EyeTribe tracker and can compute 3D coordinates from raw data and fixations. The functionality of the 3DgazeR has been tested in a case study using terrain models (DEM) as stimuli. The purpose of this test was to verify the functionality of the tool and discover suitable methods of visualizing and analyzing recorded data. Five visualization methods were proposed and evaluated: 3D raw data, 3D scanpath (fixations and saccades), 3D attention map (heat map), animation, and a graph of Z coordinate variation over time.

## Acknowledgements

A special thank you to Lucie Bartosova, who performed the testing and did a lot of work preparing data for analysis. Lukas Herman is supported by Grant No. MUNI/M/0846/2015, “Influence of cartographic visualization methods on the success of solving practical and educational spatial tasks” and Grant No. MUNI/A/ 1419/2016, “Integrated research on environmental changes in the landscape sphere of Earth II”, both awarded by Masaryk University, Czech Republic. Stanislav Popelka is supported by the Operational Program Education for Competitiveness – European Social Fund (project CZ.1.07/2.3.00/20.0170 of the Ministry of Education, Youth and Sports of the Czech Republic).
